# Supplementary material for: Selective activation of AKAP150/TRPV1 in ventrolateral periaqueductal gray GABAergic neurons facilitates conditioned place aversion in male mice
Source: Commun Biol. 2023 Jul 17;6:742. doi: 10.1038/s42003-023-05106-4 (PMC10352381; doi:10.1038/s42003-023-05106-4)

Supplementary Tables

Supplementary Table 1 Target sequence of AKAP150 and TRPV1

Gene	Target sequence
AKAP150	TCAAGAATGCTATCGAGTT
TRPV1	GCGCATCTTCTACTTCAAC

Supplementary Table 2 Adeno-associated viruses (AAV) used in the study

AAV virus	Supplier
rAAV-CaMKIIα-mCherry	Brian VTA
rAAV-CaMKIIα-hM4D(Gi)-mCherry	Brian VTA
rAAV-CaMKIIα-hM3D(Gq)-mCherry	Brian VTA
rAAV-Vgat1-mCherry	Brian VTA
rAAV-Vgat1-hM4D(Gi)-mCherry	Brian VTA
rAAV-Vgat1-hM3D(Gq)-mCherry	Brian VTA
rAAV-CMV-AKAP150-shRNA-mCherry	Brian VTA
rAAV-CMV- shRNA-NC-mCherry	Brian VTA
rAAV-CaMKIIα-AKAP150-shRNA-mCherry	Brian VTA
rAAV-CaMKIIα-shRNA-NC-mCherry	Brian VTA
rAAV-Vgat1-AKAP150-shRNA-mCherry	Brian VTA
rAAV-Vgat1-shRNA-NC-mCherry	Brian VTA
rAAV-Vgat1-TRPV1-shRNA-mCherry	Brian VTA
rAAV-Vgat1-shRNA-NC-mCherry	Brian VTA
rAAV-CMV-Cre-EGFP	Brian VTA
rAAV-CMV-EGFP	Brian VTA
rAAV-CaMKIIα-Cre-EGFP	Brian VTA
rAAV-CaMKIIα-EGFP	Brian VTA
rAAV-Vgat1-Cre-EGFP	Brian VTA
rAAV-Vgat1-EGFP	Brian VTA

Supplementary Figures

Supplementary Figure 1. CPA tests before and after AAV injection in the vlPAG in WT mice.

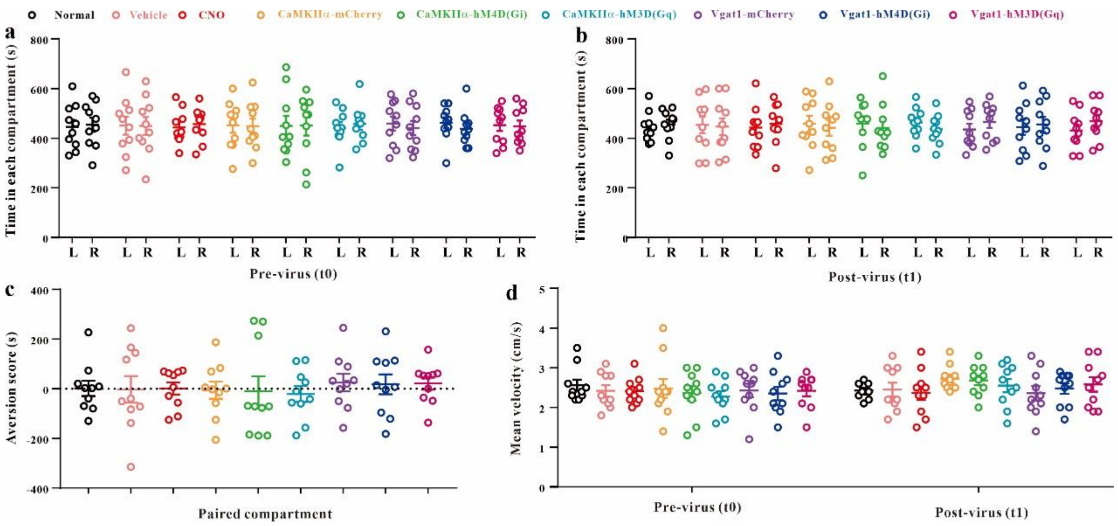

a-b. Time spent in each compartment before (t0) and after (t1) virus infection. **c.** Difference in the time (t1-t0) in the paired compartment before (t0) and after (t1) virus injection ($F_{8, 81}$ = 0.155, P = 0.996, one-way ANOVA, n = 10). **d.** Mean velocity before and after virus injection ($F_{8, 81}$ = 0.476, P = 0.870, two-way repeated-measures ANOVA, n = 10). Data are shown as the mean ± SEM.

Supplementary Figure 2. Evaluation of differences in basic preference for CPA test before and after virus injections.

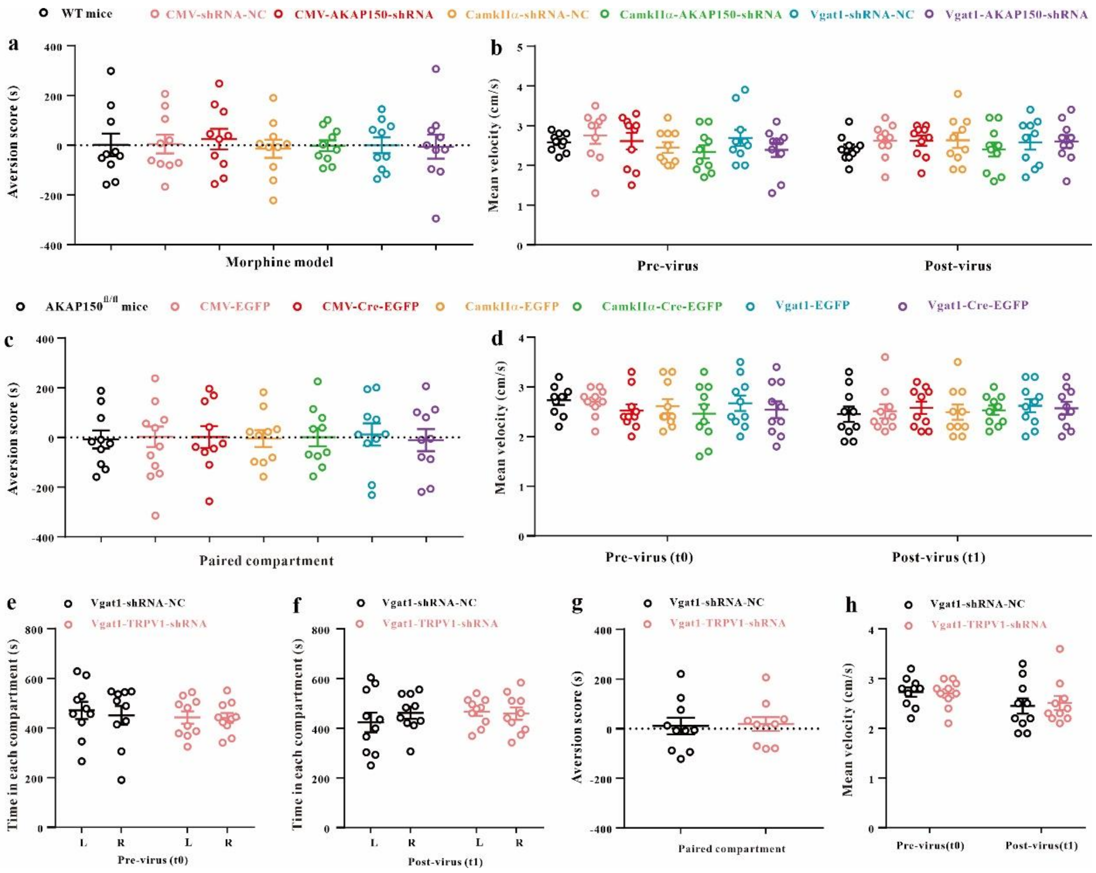

a-b. CPA tests of the WT mice with AKAP150-shRNA virus injection. Difference in the time (t1-t0) in the paired compartment before (t0) and after (t1) virus injection **(a)** ($F_{6, 63}$ = 0.097, P = 0.996, one-way ANOVA, n = 10), and mean velocity before and after virus injection **(b)** ($F_{6, 63}$ = 0.477, P = 0.823, two-way repeated-measures ANOVA, n = 10). **c-d.** CPA tests of AKAP150$^{fl/fl}$ mice injected with AAV-Cre virus injection. Difference in the time (t1-t0) in the paired compartment before (t0) and after (t1) virus injection **(c)** ($F_{6, 63}$ = 0.036, P = 0.999, one-way ANOVA, n = 10), and mean velocity before and after virus injection **(d)** ($F_{6, 63}$ = 0.488, P = 0.815, two-way repeated-measures ANOVA, n = 10). **e-h.** CPA tests of WT mice injected with Vgat1-TRPV1-shRNA. Time spent in each compartment before **(e)** and after **(f)** injection. Difference in the time (t1-t0) in the paired compartment before (t0) and after (t1) virus injection **(g)** ($F_{1, 18}$ = 0.031, P = 0.861, one-way ANOVA) and mean velocity **(h)** ($F_{1, 18}$ = 0.186, P = 0.672, two-way repeated-measures ANOVA). n = 10). Data are shown as the mean ± SEM.

Supplementary Figure 3. Raw figure data of the western blotting.

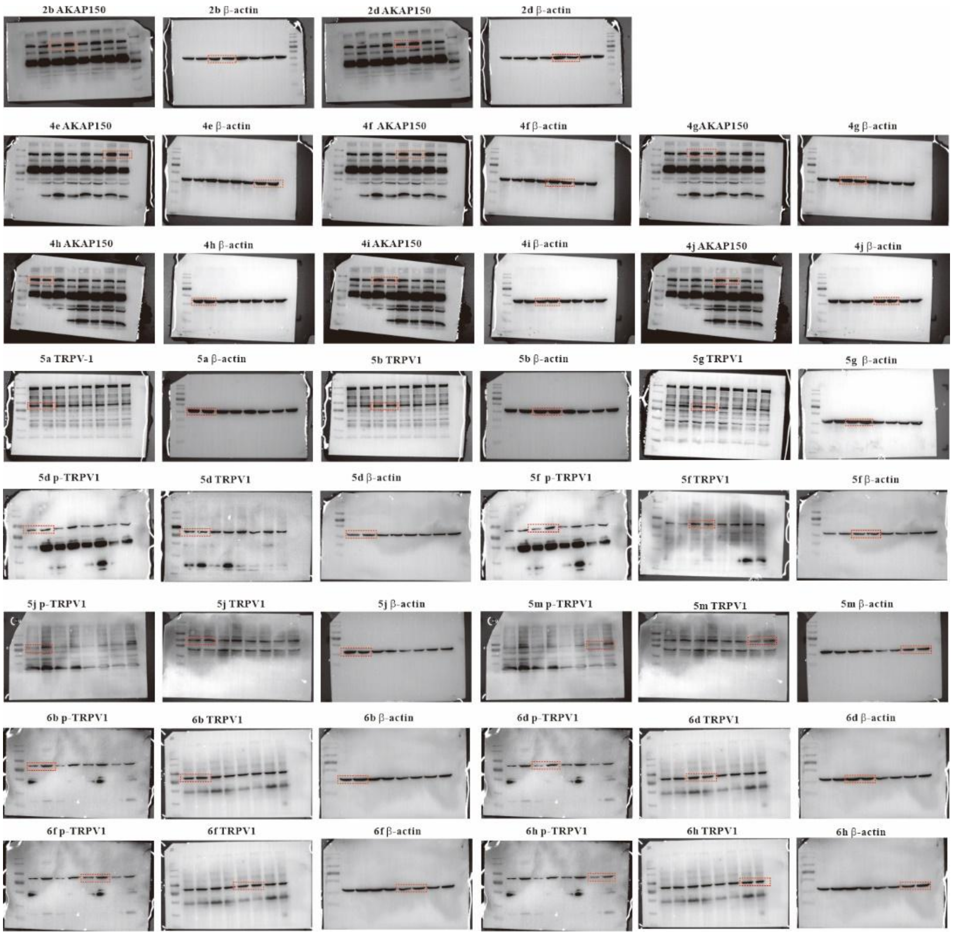

Supplement: Supplementary file 2 — Supplementary Information [file 42003_2023_5106_MOESM2_ESM.pdf]
